# Whole Gene Deletion of *EBF3* Supporting Haploinsufficiency of This Gene as a Mechanism of Neurodevelopmental Disease

**DOI:** 10.3389/fgene.2017.00143

**Published:** 2017-10-09

**Authors:** Fátima Lopes, Gabriela Soares, Miguel Gonçalves-Rocha, Jorge Pinto-Basto, Patrícia Maciel

**Affiliations:** ^1^Life and Health Sciences Research Institute (ICVS), School of Medicine, University of Minho, Braga, Portugal; ^2^PT Associate Laboratory ICVS/3B's, University of Minho, Braga, Portugal; ^3^Center for Medical Genetics Dr. Jacinto Magalhães, Centro Hospitalar do Porto, Porto, Portugal; ^4^Medical Genetics Unit, Hospital de Braga, Braga, Portugal; ^5^CGC Genetics, Porto, Portugal

**Keywords:** *EBF3*, intellectual disability, syndrome, 10qter deletion, hypotonia, movement disorder

## Abstract

Mutations in early B cell factor 3 (*EBF3*) were recently described in patients with a neurodevelopmental disorder (NDD) that includes developmental delay/intellectual disability, ataxia, hypotonia, speech impairment, strabismus, genitourinary abnormalities, and mild facial dysmorphisms. Several large 10q terminal and interstitial deletions affecting many genes and including *EBF3* have been described in the literature. However, small deletions (<1 MB) affecting almost exclusively *EBF3* are not commonly reported. We performed array comparative genomic hybridization (aCGH) (Agilent 180K) and quantitative PCR analysis in a female patient with intellectual disability. A clinical comparison between our patient and overlapping cases reported in the literature was also made. The patient carries a *de novo* 600 Kb deletion at 10q26.3 affecting the *MGMT, EBF3*, and *GLRX* genes. The patient has severe intellectual disability, language impairment, conductive hearing loss, hypotonia, vision alterations, triangular face, short stature, and behavior problems. This presentation overlaps that reported for patients carrying *EBF3* heterozygous point mutations, as well as literature reports of patients carrying large 10qter deletions. Our results and the literature review suggest that *EBF3* haploinsufficiency is a key contributor to the common aspects of the phenotype presented by patients bearing point mutations and indels in this gene, given that deletions affecting the entire gene (alone or in addition to other genes) are causative of a similar syndrome, including intellectual disability (ID) with associated neurological symptoms and particular facial dysmorphisms.

## Introduction

Intellectual disability (ID) affects nearly 1–2% of the population and is the most common neurodevelopmental disorder (NDD). A substantial number of ID patients are found to have a genetic cause (reviewed in Bessa et al., 2012). Genome-wide analysis techniques currently used for investigation of etiology often lead to the identification of very rare almost private variants, the collection of patients with alterations in the same gene being a crucial aspect of the definition of a new clinical entity.

Earlier this year, patients harboring mutations in *EBF3* gene have been described, presenting a neurodevelopmental syndrome including ID, ataxia, hypotonia, mild facial dysmorphisms, and genitourinary abnormalities (OMIM 617330) (Chao et al., 2017; Harms et al., 2017; Sleven et al., 2017). The *EBF3* (Early B cell Factor 3) gene encodes a member of the highly conserved early B-cell factor transcription factor family, expressed at high levels in the developing nervous system (data retrieved from GTEx Portal). *EBF3* is a transcriptional target of ARX, and shown to be regulated by NeuroD and ARX (Friocourt and Parnavelas, 2011). *ARX* encodes a transcription factor critical for embryonic development that, for many years, has been associated with a wide range of neurodevelopmental disorders. The intellectual impairment, central nervous system and genitourinary anomalies observed in patient with both mutations in *EBF3* and *ARX* might reflect the contribution of both proteins to the same molecular and cellular processes (Chao et al., 2017). *EBF3* function has also been studied in animal models. Ablation of its orthologs in worms and flies leads to impairment of neuronal development (Prasad et al., 1998; Hattori et al., 2013). In mice, knocking out *Ebf3* leads to neonatal lethality and neuronal migration defects, with failure of olfactory neurons project to the dorsal olfactory bulb (Wang et al., 2004). The exact pathogenic mechanisms of *EBF3* mutations is not yet fully elucidated but the type of variants described so far [copy number variations (CNVs), missense, nonsense, and splice site altering] suggest that haploinsufficiency, gain of function, and dominant negative are possible pathogenic mechanisms for the variants described (Chao et al., 2017; Sleven et al., 2017).

In this work we contribute with a patient with the smallest deletion (600 Kb) reported to date affecting the totality of *EBF3* gene and with a clinical presentation overlapping that of patients with *EBF3* single nucleotide variants (SNVs). Additionally, we make a clinical comparison of the patients with previously published large terminal 10q deletions and report that, despite the differences in size, there is a significant phenotypic overlap between patients with these alterations. These findings add to the current knowledge of *EBF3* related disorders and support *EBF3* haploinsufficiency as key in the neurodevelopmental syndrome associated with 10qter deletions.

## Materials and methods

The patient was ascertained within a large study of neurodevelopmental disorders in Portugal, in which the enrollment of the patients and families was done by the referring doctor, clinical information was gathered in an anonymous database according to the Portuguese Data Protection Authority (CNPD) and written informed consent was obtained for all participants. Informed consent for the present patient was provided by the mother for the genetic study and publication of results (including photos). The study was approved by the ethics committee of Center for Medical Genetics Dr. Jacinto Magalhães, National Health Institute Dr. Ricardo Jorge.

Genomic DNA was extracted from peripheral blood using either Citogene® DNA isolation kit (Citomed, Portugal). aCGH was performed using Agilent 180 K array (AMADID:023363) against a diploid DNA reference (Kreatech's MegaPoll Reference DNA, Kreatech Diagnostics, Amsterdam). aCGH analysis was performed using the Nexus Copy Number 6.0 software with FASST2 Segmentation algorithm (BioDiscovery Inc., El Segundo, CA). Genomic coordinates are according to Human Genome Build hg19. CNV confirmation was performed by qPCR for *EBF3* (forward primer—CTCTCTGCTGGGTGCTGAG; reverse primer—GCGTCCCTTCATACGCTAAC; ENST00000368648.7) gene and using *SDC4* (forward primer—ACCGAACCCAAGAAACTAGA; reverse primer—GTGCTGGACATTGACACCT; ENSG00000124145, Chr.20) and *ZNF80* (forward primer—GCTACCGCCAGATTCACACT; reverse primer—AATCTTCATGTGCCGGGTTA; ENSG00000174255, Chr.3) as references genes. The analysis was carried out in a 7500-FAST Real Time PCR machine (Applied Biosystems, Foster City, CA, USA) using Power SYBR Green® (Applied Biosystems, Foster City, CA, USA) according to the manufacturer's recommendations and following the general guidelines for qPCR. The specificity of each reaction was verified by the generation of a melting curve for each of the amplified fragments. The primer efficiency was calculated by the generation of a standard curve fitting the accepted normal efficiency percentage (primers used listed in Supplementary Data). Ct values obtained for each test were analyzed in DataAssist™ software (Applied Biosystems, Foster City, CA, USA).

## Results

Here we describe a patient with a *de novo* deletion affecting *EBF3*. The patient is an 11 years old girl with severe ID (global development quotient = 27 at 7 years of age), born from non-consanguineous parents and with no family history of neurodevelopmental disorders. She was born after a biamniotic bichorionic twin pregnancy (her sister being healthy), by vaginal delivery, at 35 weeks of gestation. Birth parameters were: weight, 1,830 g (P3); length, 42.5 cm (P10); and OFC, 30.6 cm (P10), with an Apgar score of 8/9 (1st and 5th min, respectively). The neonatal period was complicated with sepsis and the diagnosis of hereditary spherocytosis (inherited from her mother). Global developmental delay was noted in the first months, with head control achieved at 12 months, sitting at 18 months, independent walking at 30 months, and no words spoken at the age of 3 years. She had pyelonephritis at 19 months (renal ultrasound showed no abnormalities), gastroesophageal reflux and recurrent otitis media, with conductive hearing loss that required surgical intervention and a hearing aid. Epilepsy was suspected at 5 months (episodes of suspended activity) but the EEG was normal.

**Figure 1:**
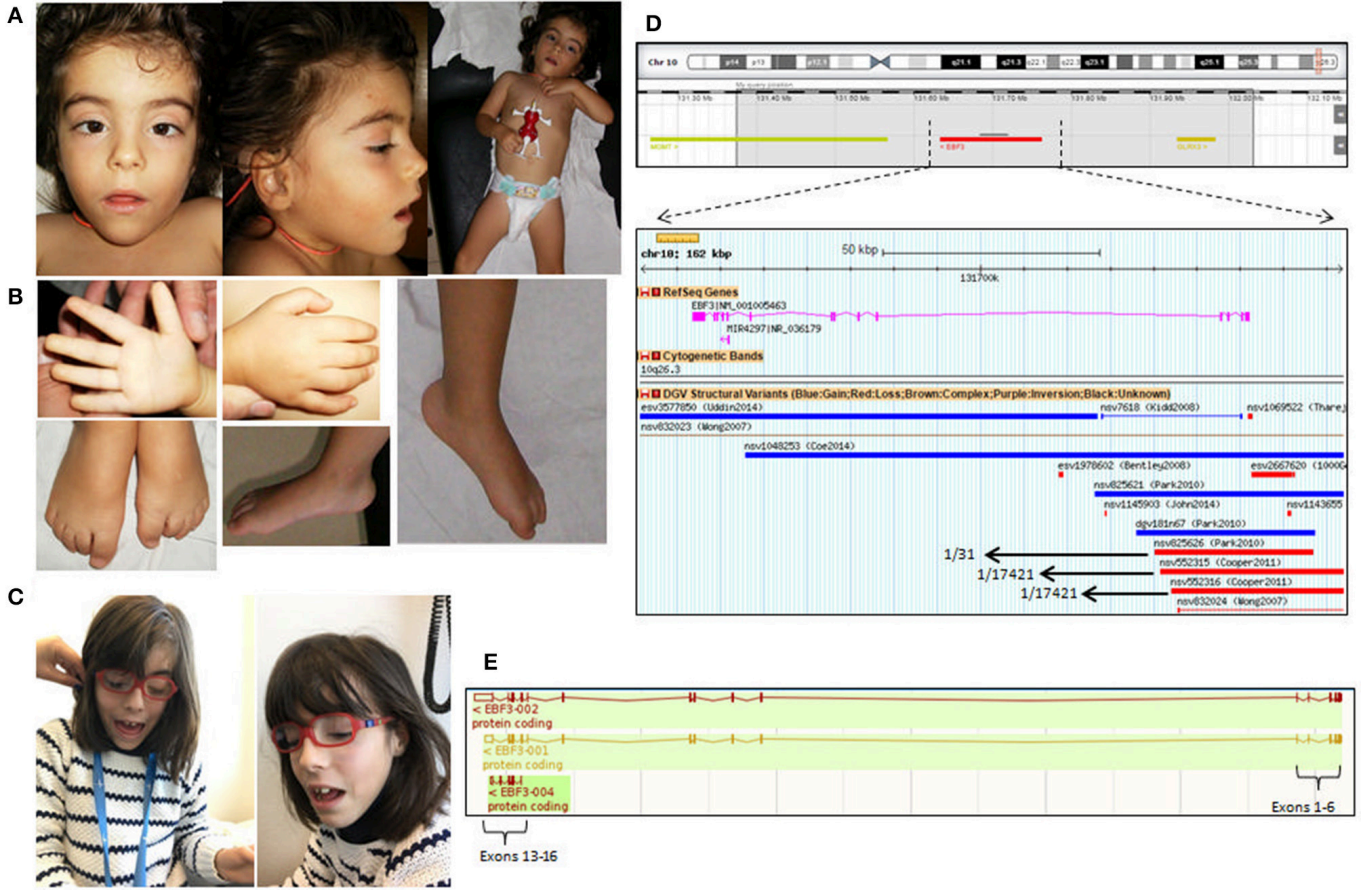
**(A)** Facial appearance of the patient at 3 years and 5 months showing the small and low-set ears with prominent anti-helix and **(B)** fetal pads in the fingers. **(C)** Facial appearance of the patient at 11 years of age. **(D)** Highlighted in gray the 600 Kb deletion at 10q26.3 region; a zoom in of the *EBF3* gene in the DGV database reveals the existence of 3 deletions in 3 controls that affect the first 6 exons of *EBF3* (NM_001005463); CNVs within this region found in control populations include deletions nvs825626 (present in 1/31 individuals), nvs552315 (present in 1/17421 individuals) and nsv552316 (present in 1/31 individuals). **(E)** The schematic representation of *EBF3* transcripts.

She was first observed at the age of 3 years 5 months (Figures 1A,B), at which time she displayed muscle hypotonia, hypotonic face, strabismus, and reduced sensitivity to pain. She also presented mild dysmorphic features (Figure 1A): triangular face, small low-set ears with prominent anti-helix, arched eyebrows, anteverted nares, bulbous nasal tip, small mouth with downturned corners, pointed chin, short neck, and prominent finger fetal pads, as well as a mild short stature (89 cm, corresponding to around 2SD).

Brain MRI was performed at 6 years, but no abnormalities were noted. At the age of 10 years she was reevaluated; she still had recurrent otitis media, but otherwise was in good global health. Language was very poor (two word sentences spoken after 8 years). She had behavior problems, with stereotypic movements (rotating movements, chewing on clothes, head retropulsion), scoring for severe autism spectrum disorder (ADI-R and ADOS) at 7 years; she displayed agitation and aggressive behavior (auto and hetero) and was medicated with antipsychotic drugs. An orthopedic surgery was performed for *pes planus*. The facial features were similar to those previously described, with spaced upper central incisors (Figure 1C); she had eyeglasses for strabismus and hypermetropia.

Analysis of genomic DNA by aCGH revealed a *de novo* 600 kb deletion at 10p26.3 (Figure 1D) affecting three genes—*MGMT* (encoding the enzyme O-6-methylguanine-DNA methyltransferase, involved in DNA repair), *EBF3* and *GLRX* (encoding glutaredoxin, a small thioltransferase that removes protein GSH adducts), of which *EBF3* was the most likely disease-associated gene.

## Discussion

The presented patient was first analyzed by aCGH a few years ago. At the time of aCGH analysis, the existence of the three variants present in Database of Genomic Variants (DGV)^1^ affecting the first five exons of *EBF3* gene (Figure 1E; Park et al., 2010; Cooper et al., 2011), as well as the absence of other known disease causing mutations in this gene, lead us to classify it as a variant of unknown significance (VOUS). However, the recent publications of *EBF3* mutations (Chao et al., 2017; Harms et al., 2017; Sleven et al., 2017) and the clinical similarities with the reported cases, lead us to re-assess the variant and make us believe that *EBF3* deletion may in fact be accounting for the disease in the patient. One of the aspects that raised doubts about the pathogenicity of this variant in the first place was the existence of population controls bearing deletions of the first six exons of this gene, in heterozygosity (data retrieved from DGV database as of February 2017) (Figure 1D). Even though a transcript of *EBF3* starting in Exon13b is listed in the Ensembl database (ENST00000440978.1) (Figure 1E), which could explain how deletion of the first exons could eventually result in a normal phenotype, this transcript excludes the DNA binding domain of EBF3, and its expression pattern and functional relevance have not been characterized. Upon reassessment, however, the CNVs described by Cooper and colleagues (nsv552315 and nsv552316) (Chao et al., 2017; Harms et al., 2017; Sleven et al., 2017) were considered to be at the threshold of detection by SNP microarray and cannot be the basis for exclusion of a candidate gene, particularly in light of the strong genetic and functional evidence for the relevance of *EBF3* mutations to disease (Evan Eichler, Greg Cooper and Bradley Coe, personal communication).

Our patient shows many clinical similarities with previously described patients with mutations in *EBF3* (21 cases summarized in Table 1), such as global developmental delay, delayed expressive speech, hypotonia, increased pain threshold, behavioral problems and characteristic facial features (long/triangular face, large forehead, hypotonic face). However, even though our patient had significant delay in motor development, no significant ataxia was detected (with some limitations in clinical examination, as the child was not cooperative), and no cerebellar anomalies were present in brain MRI.

**Table 1:** Clinical comparison of the present case with the reported cases with point mutations/indels in the *EBF3* gene.

		**Harms et al**.										**Chao et al**.			**Sleven et al**.								**%**	**Freq**.
	**Our case**	**P1**	**P2**	**P3**	**P4**	**P5**	**P6**	**P7**	**P8**	**P9**	**P10**	**P1**	**P2**	**P3**	**P1**	**P2**	**P3**	**P4**	**P5**	**P6**	**P7**	**P8**		
**Mutation**	**Del**	**Miss**.	**Miss**.	**STOP**	**Miss**.	**Spl**.	**Miss**.	**Miss**.	**Miss**.	**STOP**	**InDel**	**Miss**.	**Miss**.	**Miss**.	**Miss**.	**Miss**.	**Spl**.	**Miss**.	**FrShf**.	**Spl**.	**STOP**	**STOP**		
Global developmental delay	Y	Y	Y	Y	Y	Y	Y	Y	Y	Y	Y	Y	Y	Y	Y	Y	Y	Y	Y	Y	Y	Y	100	21/21
ID	Y	Y	Y	Y	Y	Y	Y	Y	Y	Y	Y	Y	Y	Y	Y	Y	Y	Y	Y	Y	NI	NI	100	19/19
Recurrent infections	Y										Y								Y	Y			14	3/21
Gastroesophageal reflux	Y				Y							Y											10	2/21
Conductive hearing loss	Y																						0	0/21
Strabismus	Y	Y	Y	Y	Y	Y	Y	Y	Y		Y	Y	Y		Y	Y	Y	Y	Y	Y			81	17/21
Hypermetropia	Y																						0	0/21
Muscle hypotonia	Y	N	N	N	Y	NA	Y	Y	Y	Y	N	Y	Y	Y	Y	Y	Y	Y	Y	Y	Y	Y	80	16/20
Reduced pain sensitivity	Y	NI	NI	NI	NI	NI	NI	NI	NI	NI	NI		Y	Y		Y							27	3/11
Facial dysmorphisms	Y	Y	Y		Y	NA	NA		Y	Y		Y	Y	Y	Y	Y	Y	Y	Y		Y	Y	79	15/19
Hypotonic face	Y								Y			Y	Y	Y									19	4/21
Triangular face	Y												Y										5	1/21
Dysmorphic ears	Y	Y	Y	N	Y	NA	NA	N	N	NA	Y	Y	Y				Y						56	10/18
Arched eyebrows	Y																						0	0/21
Anteverted nares	Y																						0	0/21
Bulbous nose	Y																Y						5	1/21
Small mouth	Y			Y													Y						10	2/21
Pointed chin	Y								Y														5	1/21
Short neck	Y																	Y					5	1/21
Finger fetal pads	Y																						0	0/21
Short stature	Y														Y		Y	Y	Y				19	4/21
Pes planus	Y														Y								5	1/21
Language delay	Y	Y	Y	Y	Y	Y	Y	Y	Y	Y	Y	Y	Y	Y	Y	Y	Y	Y	Y	Y	Y	Y	100	21/21
Behavior problems	Y				Y								Y			Y	Y	Y			Y	Y	33	7/21
Stereotypic movements	Y																						0	0/21
Autism	Y																						0	0/21
Agitation	Y																						0	0/21
Agressive behavior	Y																						0	0/21

*Del, Deletion; Miss, Missense; Spl, Splice Site; FrShf, Frame Shift; Y, Yes; N, Normal; NI, No information; NA, Not available*.

Several large terminal 10q26 deletions have been reported in the literature (24 cases, summarized in Table 2; Turleau et al., 1979; Evans-Jones et al., 1983; Zatterale et al., 1983; Shapiro et al., 1985; Mehta et al., 1987; Gorinati et al., 1989; Wulfsberg et al., 1989; Kogasaka et al., 1990; Schrander-Stumpel et al., 1991; Wilkie et al., 1993; Petit et al., 1998; Leonard et al., 1999; Waggoner et al., 1999; Lukusa et al., 2002; Iourov et al., 2014) and in Decipher (60 cases), in patients who share some clinical features with our patient, such as developmental delay and/or ID (present in all cases in which the patient was old enough to evaluate), short stature (10/24), hypotonia (12/24), strabismus (13/24), triangular facial appearance (9/24), and dysmorphic ears (14/24). Even though these patients have much larger deletions, the similarities suggest that *EBF3* may also be an important contributor to their phenotype.

**Table 2:** Clinical comparison of the present case with the reported cases with deletions affecting the 10q26 cytoband.

	**Our case**	**Turleau 1979**	**Evans Jones 1983**	**Zatterle 1983**	**Shapiro 1985 P1**	**Shapiro 1985 P2**	**Shapiro 1985 P3**	**Mehta 1987**	**Gorinati 1989**	**Wulfsberg 1989 P1**	**Wulfsberg 1989 P2**	**Wulfsberg 1989 P3**	**Kogasaka 1990**	**Wilkie 1993 P1**	**Wilkie 1993 P2**	**Schrander-Stumpel 1994 P1**	**Schrander-Stumpel 1994 P2**	**Petit 1998 P1**	**Petit 1998 P2**	**Leonard 1999**	**Waggoner 1999 P1**	**Waggoner 1999 P2 (q25.3q26.3)**	**Waggoner 1999 P3**	**Lukusa 2000**	**Iourov 2014**	**%**	**Freq**.
**Deletion breakpoint**	**q26.3**	**q26**	**q26.2**	**q26.1**	**q26**	**q26**	**q26**	**q26.1**	**q26**	**q25.3**	**q26.1**	**q26**	**q26.1**	**q26.1**	**der(10) t(10;16) (q26.2;q21)**	**q25.3**	**q26**	**q26**	**q26**	**q26.1**	**q26.2**	**q25.3q26.3**	**q26.1**	**q26.3**	**q26.2**		
Global developmental delay	Y	Y	Y	Y	Y	Y	Y	Y	Y	NP	Y	NP	Y	Y	Y	Y	Y	Y	Y	Y	Y	Y	Y	Y	Y	100	22/22
ID	Y	Y	Y		Y	Y	Y	Y	Y	NP		NP	Y	Y		Y	Y	Y	Y	Y			Y	Y		68	15/22
Recurrent infections	Y								Y								Y			Y							
Gastroesophageal reflux	Y																			Y						4	1/24
Conductive hearing loss	Y							Y			Y															8	2/24
Strabismus	Y	Y	Y				Y	Y	Y		Y				Y	Y	Y		Y	Y			Y	Y		54	13/24
Hypermetropia	Y																										
Muscle hypotonia	Y		Y		Y						Y		Y	Y			Y		Y	Y	Y	Y	Y	Y		50	12/24
Reduced pain sensitivity	Y																										
Facial dysmorphisms	Y			Y						Y	Y	Y		Y	Y	Y	Y			Y	Y	Y	Y	Y	Y	58	14/24
Hypotonic face	Y																										
Triangular face	Y	Y	Y	Y				Y	Y						Y					Y	Y			Y		38	9/24
Dysmorphic ears	Y		Y	Y		Y			Y	Y	Y	Y	Y			Y	Y			Y	Y		Y		Y	58	14/24
Arched eyebrows	Y																										
Anteverted nares	Y								Y													Y				8	2/24
Bulbous nose	Y							Y								Y										8	2/24
Small mouth	Y																					Y				4	1/24
Pointed chin	Y							Y																		4	1/24
Short neck	Y	Y		Y					Y												Y		Y			21	5/24
Finger fetal pads	Y																Y									4	1/24
Short stature	Y		Y	Y				Y			Y			Y		Y	Y				Y	Y	Y			42	10/24
Pes planus	Y																								Y	4	1/24
Language delay	Y							Y						Y		Y	Y			Y				Y		25	6/24
Behavior problems	Y							Y								Y	Y	Y			Y			Y		25	6/24
Stereotypic movements	Y																							Y		4	1/24
Autism	Y																										
Agitation	Y							Y										Y			Y			Y		17	4/24
Agressive behavior	Y							Y													Y					8	2/24
Others										Died at 1 month		Epilepsy; died at 3 weeks		Genital anomalies	Genital anomalies					Several urinary tract problems				Cryptorchidism, hypogenitalism			

*Y, Yes; NP, Not possible to determine*.

The *EBF3* variants described in the literature in the beginning of 2017 include point mutations predicted to be deleterious and small insertions and deletions leading to in frame deletion of key aminoacids or to a frameshift, predictably causing early truncation of the resulting protein or nonsense-mediated decay. The mutations were concentrated on parts of the gene encoding the DNA-binding domain of EBF3, and were predicted through different methods to lead to a loss of function of this transcription factor, thus suggesting reduced function and haploinsufficiency as the mechanism underlying the neurodevelopmental disturbance in these patients. Knock-out mice for *Ebf3* are described to present neonatal lethality and neuronal migration defects, with failure of olfactory neurons to project to the dorsal olfactory bulb (Wang et al., 2004), but no description is made of a phenotype in the heterozygous animals, which are actually presented as controls in many of the experiments, thus not supporting the haploinsufficiency model. We made efforts to obtain and study the neurodevelopmental phenotype of these animals, but were not successful, as the *Ebf3(O/E2)* knock-out line may have been discontinued (Joseph W. Lewcock, personal communication). However, the current case together with the patients summarized in Table 2, do support the hypothesis of *EBF3* haploinsufficiency as disease causing.

In summary, the current description reinforces *EBF3* loss of function/haploinsufficiency as a cause of neurodevelopmental disease, and reinforces the association of this gene with a characteristic clinical syndrome within this spectrum.

## Author contributions

FL performed the molecular studies and analyzed the molecular data. MG and GS collected clinical data. FL, JP, and PM reviewed all *EBF3* mutation cases in the literature. FL, GS, JP, and PM drafted the paper. PM obtained funding for this study. The study was performed under the direction of PM.

### Conflict of interest statement

JP was employed by company CGC Genetics. The other authors declare that the research was conducted in the absence of any commercial or financial relationships that could be construed as a potential conflict of interest.

## Footnotes

^1^Database of Genomic Variants Available at: http://dgv.tcag.ca/dgv/app/home [Accessed August 5, 2014].
